# The challenges of getting the research published when English is not the first language: the example of Mozambique Field Epidemiology Training Program

**DOI:** 10.11604/pamj.2019.33.208.18766

**Published:** 2019-07-16

**Authors:** Cynthia Semá Baltazar, Caroline Wheatley, Peter Nsubuga

**Affiliations:** 1 National Institute of Health, Maputo, Mozambique; 2 Wheatley Dias Consulting, LDA, Maputo, Mozambique; 3 Global Public Health Solutions LLC, Altanta, USA

**Keywords:** Challenges, published, non-English, Mozambique

## Abstract

The Mozambican Field Epidemiology and Laboratory Training Program (Moz-FELTP) is a two-year, competency-based post-graduate training and service program designed to build sustainable public health capacity in applied epidemiology. Despite the efforts, Moz-FELTP residents have historically difficulty to publishing their work for a variety of reasons that includes language barriers, lack of writing skills and motivation, limited budgetary support and lack of effective mentorship. This outline the need for different approaches to continuous improving the publication, such scientific writing mentorship for non-English FELTP residents.

## To the editors of the Pan African Medical Journal

The Moz-FELTP is a post-graduate (master) residency program that combines classroom and applied training with mentored in-service activities to train public health professionals in applied epidemiology and disease control [1]. Moz-FELTP residents are taught in Portuguese which is the official language in Mozambique, and they graduate from Universities in Mozambique. Moz-FELTP residents conduct outbreak investigations, surveillance system evaluations, data analysis, and final thesis research, all of which can be published and would add value to the broader public health community. Implementation of the Moz-FELTP is in its seventh year, and it has successfully trained 46 public health professionals in the 2-year advanced course. Residents acquire an array of practical public health skills in disease surveillance and outbreak investigation, data management, monitoring and evaluation of health programs, scientific writing and communication [2]. However, very few articles from residents have been published, disseminated and communicated to the broader public. This is primarily due to the challenges non-native English speakers face in submitting their articles for publication and that most of the residents have never published a peer-reviewed article before starting the master program. Other reasons could also include a lack of time or low priority for scientific publications.

Scientific publication is one of the most important means for communicating in the scientific world and is an essential metric for scientific careers. It has been increasingly recognized as a responsibility of scientists, where the ability to accurately and effectively communicate ideas, procedures, and findings according to readers' expectations are the primary skills required for scientific writing [3]. Publishing is fundamental to disseminate new knowledge and developed evidence-based policies with the ultimate goal to improve public health [4]. The scientific community is highly demanding regarding the dissemination of research results and scientific publications need to be written with high accuracy to be accepted in scientific journals [5]. Despite the importance of publishing scientific articles for knowledge dissemination and career success, publication privileges native English speakers, as most of the current public health journals are in english. There are many challenges that non-native English speakers face, particularly those from developing countries like Mozambique. Some of these challenges include access to reference articles, the ability to effectively communicate research in a non-native language, the lack of journals and visibility in researchers' native languages, the cost of publication and the lower indexing given to national journals that publish in native languages.

Non-English-speaking authors are at an even more significant disadvantage since their language limits them to 'sell' their work. The highest-ranking journals are in English, which creates an additional impediment for non-native English speakers who need to reference these articles in their manuscripts. For visibility and ranking of published papers, authors are encouraged to publish in highly rated and indexed biomedical journals [5]. Given the history of the low level of publications by Moz-FELTP residents and the related challenges of publishing in highly rated journals, publishing in non-indexed local magazines is an alternative for residents. This does not necessarily mean the quality of the research is poor but is an indication of the challenges of publishing. The lack of access to subscription-based scientific journals inhibits the exposure of many scientists from developing countries, including Mozambique, to use the most up-to-date research knowledge to strengthen their research. Usually, residents do not have the funding or do not know how to access these articles freely and therefore rely on Open Access Journals which help to level the playing field for resource limited programs [6]. An additional challenge is the expenses that are often required by the authors to publish their articles. Due to this, authors from resource-limited countries are limited to journals that waive the fee for authors from low-income countries, and therefore our residents do not have the same access to publication as authors from higher income countries. One further challenge the residents have is learning how to write for a global audience. In the Moz-FELTP program, not all residents are fluent in English which may limit understanding of current research and their ability to compare their results with those in other countries. Residents have also been at a disadvantage when presenting at international conferences during the question and answer time after their presentations, these question and answer periods improves the quality of the final research report. For many residents, their English is acceptable when giving a prepared presentation, but they face difficulty being able to respond to questions and explain the intricacies of their research.

Strong scientific writing skills also condition public health researchers to be successful in presenting proposed studies for approval and funding. Despite the importance of publishing, scientific writing is a difficult skill that many researchers, especially the youngest ones often lack in the African context [5]. Most younger scientists are not able to meet the high standards of international journals due to the paucity of resources, infrastructure and training to build the specific skills necessary for scientific writing [5, 7]. One of the most common outcome measures for a training programs is trainee publication rate. However, there has been little discussion of how to weigh publications, including the relative value of peer-reviewed versus non-peer reviewed publications and first-author versus other-author publications and there are few considerations to the extensive process non-native English speakers encounter when submitting a manuscript for publication in an international peer-reviewed journal [8]. To overcome some of these obstacles, the increased and repeated exposure in international scientific events could allow Moz-FELTP to write better manuscripts and increases their chances of becoming published. Participating in a conference, submitting their first article, or even seeing their peers accomplish these goals, makes authorship and high academic achievement seem achievable and expected. Writing a manuscript for an international journal or attending an international conference emphasizes the necessity of strong English language skills. While all Moz-FELTP residents received English training at least from secondary school, some have cultivated their language skills more than others. A Scientific Writer Program can improve their skills and is more likely to generate a more globally minded and refined cohort of residents who are better positioned to showcase their research throughout their careers. Residents mentored are more likely to have completed manuscript reviews compared to the non-mentored. It has been found that mentoring promotes the scholastic development of authors, improves writing skills, and provides personal support [9-11].

## Conclusion

Reducing the language barrier for non-English speaking scientists will not only benefit these scientists, their careers and their training programs, but it will greatly benefit the scientific community. In the case of the Moz-FELTP residents, they respond to numerous outbreaks and monitor and evaluate prevention and control efforts for communicable diseases such as TB and HIV. Their experiences are vital for the international community. Therefore, efforts to reduce language barriers, ensure equitable access to journal articles and increase publication of research from non-English speaking and resource limited countries is required.

## Competing interests

The authors declare no competing interest.
